# VDJviz: a versatile browser for immunogenomics data

**DOI:** 10.1186/s12864-016-2799-7

**Published:** 2016-06-13

**Authors:** Dmitriy V. Bagaev, Ivan V. Zvyagin, Ekaterina V. Putintseva, Mark Izraelson, Olga V. Britanova, Dmitriy M. Chudakov, Mikhail Shugay

**Affiliations:** Shemyakin-Ovchinnikov Institute of Bioorganic Chemistry RAS, Miklukho-Maklaya 16/10, 117997 Moscow, Russia; Pirogov Russian National Research Medical University, Ostrovityanova 1, 117997 Moscow, Russia; Central European Institute of Technology, Masaryk University, Brno, Czech republic

**Keywords:** High-throughput sequencing, Immunology, Browser, T-cell, B-cell, Repertoire sequencing

## Abstract

**Background:**

The repertoire of T- and B-cell receptor sequences encodes the antigen specificity of adaptive immunity system, determines its present state and guides its ability to mount effective response against encountered antigens in future. High throughput sequencing of immune repertoires (Rep-Seq) is a promising technique that allows to profile millions of antigen receptors of an individual in a single experiment. While a substantial number of tools for mapping and assembling Rep-Seq data were published recently, the field still lacks an intuitive and flexible tool that can be used by researchers with little or no computational background for in-depth analysis of immune repertoire profiles.

**Results:**

Here we report VDJviz, a web tool that can be used to browse, analyze and perform quality control of Rep-Seq results generated by various pre-processing software. On a set of real data examples we show that VDJviz can be used to explore key repertoire characteristics such as spectratype, repertoire clonality, V-(D)-J recombination patterns and to identify shared clonotypes. We also demonstrate the utility of VDJviz in detection of critical Rep-Seq biases such as artificial repertoire diversity and cross-sample contamination.

**Conclusions:**

VDJviz is a versatile and lightweight tool that can be easily employed by biologists, immunologists and immunogeneticists for routine analysis and quality control of Rep-Seq data. The software is freely available for non-commercial purposes, and can be downloaded from: https://github.com/antigenomics/vdjviz.

**Electronic supplementary material:**

The online version of this article (doi:10.1186/s12864-016-2799-7) contains supplementary material, which is available to authorized users.

## Background

A diverse repertoire of T- and B-cell antigen receptors is a critical component of host defense system in vertebrates called adaptive immunity which ensures readiness and ability to detect and mount an effective response against the great variety of encountered pathogens. T- and B-cell receptor repertoire is formed by genomic rearrangement of Variable (V), Diversity (D) and Joining (J) segment loci in a process called V-(D)-J recombination [1]. Each of the resulting segment junctions carry complementarity determining region 3 (CDR3) that plays a key role in antigen recognition and largely defines the specificity of T-cells and immunoglobulins [2]. Recent advances in molecular methods and high-throughput sequencing allow to profile antigen receptor repertoires using a technique called Rep-Seq [3]. Raw receptor sequences produced by Rep-Seq can be processed by one of the existing bioinformatics software tools (http://omictools.com/rep-seq-c424-p1.html) to map V-(D)-J junctions and extract CDR3 regions, thus forming a set of clonotypes - unique combinations of V, D and J segments, and CDR3 sequence. Those mappings are then assembled to estimate individual clonotype frequencies that reflect clonal expansions caused by antigen recognition, peripheral selection [4] and convergent V-(D)-J recombination processes [5]. Resulting datasets are inherently complex due to extremely high diversity of T- and B-cell receptor sequences and a plethora of physiological factors that shape the repertoire structure [6].

Rep-Seq technique has the potential to become a method of choice for biologists studying adaptive immunity [7], however the software framework behind this field is still relatively immature. Importantly, this field is in need of tools that can be used by biologists with little or no computational knowledge: while there is a substantial number of tools dedicated to data processing [8–15], there is a considerable lack of options to analyze resulting immune repertoire profiles. In order to fill this critical gap we have developed VDJviz, an open-source web-based graphical user interface (GUI) software for Rep-Seq data browsing. Main features of VDJviz can be summarized as follows:

- A parser that supports output from 6 commonly used Rep-Seq processing software: MiTCR, MIGEC, MiXCR, IgBlast, IMGT HighVQuest and ImmunoSEQ platform; as well as an internal concise tab-delimited format.
- An intuitive clonotype table viewer with V-(D)-J markup that can be used to navigate through the entire sample and perform complex searches.
- Comprehensive single sample analysis modules calculating basic repertoire statistics and providing interactive spectratype, V/J segment usage and clonality plots.
- Extended multi-sample analysis that includes clonotype tracking and sample intersection with a flexible set of clonotype matching rules, repertoire diversity comparison using rarefaction and simple side-by-side comparison of single sample analysis results.
- Export of analysis results and dataset sharing.

## Implementation

VDJviz is a web based GUI application that uses VDJtools API (https://github.com/mikessh/vdjtools) as a back-end. The software utilizes Play framework (https://www.playframework.com/) for running the server instance and state-of-art web graphics libraries such as D3js (http://d3js.org/) for visualization. The reason for choosing Play framework is its stability and ease of deployment, while D3js allows us to create complex interactive plots. The browser is lightweight and uses around 4GM RAM to host several users analyzing 25 samples of up to 10,000 clonotypes, which is the upload limit for the demo version available online. This limit can be removed for local installations to allow browsing large samples using better hardware. In most cases users can also down-sample clonotype abundance tables (http://vdjtools-doc.readthedocs.org/en/latest/preprocess.html#downsample) to view large samples with commodity hardware.

## Results and discussion

To the best of our knowledge, in contrast to the rich set browsers available in the field of genomics (e.g. Refs. [16–18]), the only published software that falls in immune repertoire browser category so far is IMEX [19]. Existing unpublished solutions for immune repertoire browsing include VDJserver (https://vdjserver.org/), Vidjil browser (http://www.vidjil.org/#browser) and ImmunoSEQ Analyzer (https://clients.adaptivebiotech.com/). In this section we will first compare the functionality of VDJviz with aforementioned web tools and demonstrate VDJviz features on the set of relevant examples further in the text.

IMEX is a closed-source GUI software that allows computing basic repertoire statistics, analyzing V-D-J segment usage, performing diversity estimation and provides some options for comparing clonotype tables. The software is implemented using .NET technology and can natively run on Windows. Running it on Unix-based systems requires setting up the Mono Framework (http://www.mono-project.com/). There are several general limitations of IMEX compared to VDJviz. First, IMEX limits its analysis to datasets produced by IMGT High-V/Quest software while VDJviz allows both IMGT High-V/Quest input and input generated by other software tools. IMGT High-V/Quest is frequently used by immunologists, however the current upload batch size of 0.5 mln reads and variable submission/processing times makes it unfeasible for analysis of large datasets containing tens of millions of reads. Next, currently IMEX is limited to TRB and IGH loci while no such limitation exists in VDJviz. However the most important issue with IMEX is the way it estimates one of the key immune repertoire parameters, repertoire diversity [20]. IMEX fits the *a × (1-exp(−b × n)) + k × n* function, where *n* is sampling depth, *a* is real number of clonotypes and *k* is the error rate, using an optimization algorithm to the rarefaction curve obtained by random re-sampling. This empirical model can produce spurious results in some common settings and cannot reliably distinguish rare clonotypes and errors. For example, let us consider a highly diverse and uniform repertoire (say, naive T-cells) and note that corresponding Rep-Seq data can have negligible error rate if produced using high-fidelity protocols [12, 21]. The rarefaction curve in error-free setting is a linear function of sample size [22]. On the other hand, the optimal parameters for model used in IMEX can be selected as k = 1 and a = 0/b = any or b = 0/a = any in this setting, thus either rendering all clonotypes as erroneous or providing an arbitrary number of clonotypes in a sample that depends on the seed of the random number generator used by the optimization algorithm. VDJviz, on the other hand implements a robust and commonly used rarefaction algorithm [22] leaving the choice of error correction strategy up to the user.

Vidjill browser is an extension of recently published Vidjil Rep-Seq processing software [23]. The major difference between Vidjil browser and VDJviz lies in the repertoire browsing implementation and repertoire analysis features. Vidjil browser operates with V-D-J signatures of clonotype clusters and implements a graphical clonotype tracking interface with an aim to facilitate clonotype tracking for MRD detection and monitoring. VDJviz, on the other hand, lists individual clonotypes in tabular format and all the relevant information such as V,D and J segments and the CDR3 region sequence, which allows to directly browse the clonal composition of sample and perform clonotype table searches using pattern-matching and filters. Notably, VDJviz implements some of the commonly used analysis modes such as diversity estimation and spectratyping that are not present in Vidjil browser. VDJviz also implements basic clonotype tracking functionality in its cross-sample intersection and clonotype search modules. VDJviz doesn’t limit clonotype tracking to samples coming from the same donor, allowing to match clonotypes based on CDR3 amino acid sequences and therefore allows exploring clonotypes shared by several different donors.

VDJserver software, being in beta version, incorporates V-D-J mapping engine and requires to upload raw sequencing data, which can be both considered as a benefit and a limitation comparing to VDJviz that accepts processed data in multiple formats. While doing data processing on server side facilitates analysis for data produced using common library preparation protocols, it is unfeasible to implement a general algorithm that covers all possible customizations of those protocols and complex cases such as multiplexing and unique molecular identifier tagging [12]. The output provided by VDJtools includes segment usage chart and V-D-J mapping statistics, while clonotype tables are only available as a downloadable plain text file, which is far less than the functionality provided by VDJviz, Vidjil browser and IMEX.

ImmunoSEQ analyzer is a commercial software and supports only customer data produced by corresponding commercial assay. ImmunoSEQ has a rich feature set, some of which are not present in VDJviz, namely a variety scatterplots for sample comparison, immunoglobulin somatic hypermutation and edit distance analysis. VDJviz, on the other hand, offers more options for diversity estimation including rarefaction analysis and clonality plot, clonotype-level detalization for sample intersection and a powerful clonotype search engine. Clonotype search algorithms of VDJviz are also more flexible: various filters such as segment filter can be used in combination, user can search for CDR3 sequence patterns and several clonotype matching modes are supported, for example amino acid-not-nucleotide matching that can be used to filter cross-sample contaminations.

Below we present six example cases that demonstrate the usability of VDJviz for common immune repertoire analysis tasks, in-depth browsing of repertoire clonal composition and detection of Rep-Seq artifacts. Data for reproducing all the examples presented here is available in the “examples” folder of VDJviz source code repository, all figures in this paper are screenshots of VDJviz browser interface.

### Example 1: spectratyping

**Fig. 1:**
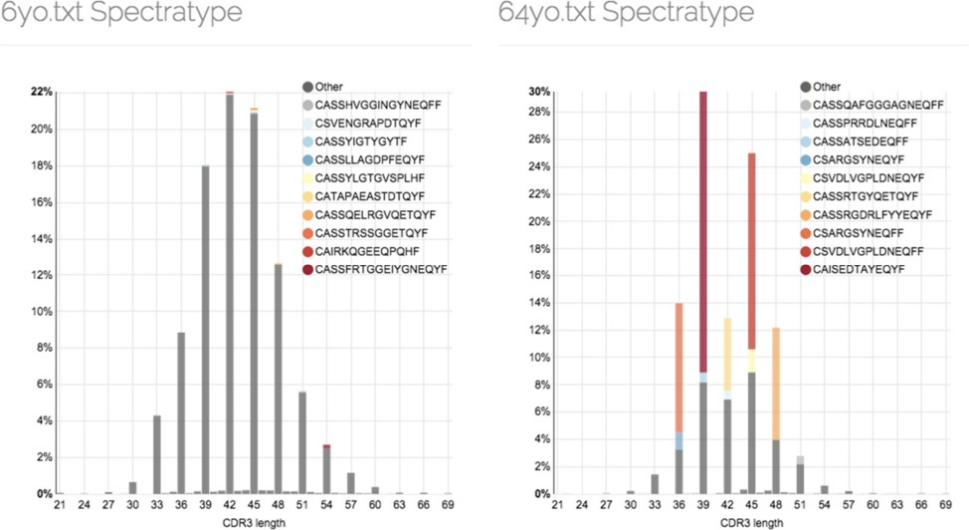
Spectratypes of 6 and 64 years old donors. Each bin of the histograms corresponds to the length of CDR3 nucleotide sequence, clonotypes were weighted by their frequency and the fraction of top 10 most abundant clonotypes is shown with *colored bars*. Note that *bars* at CDR3 lengths that are not a multiple of 3 represent out-of-frame clonotypes

The first example demonstrates a variation of conventional spectratype (the distribution of CDR3 lengths) analysis that also visualizes the most abundant clonotypes. Repertoires of 6 and 64 years old healthy donors from our aging study [24] were analyzed. Those samples were prepared using a protocol that allows accurate TCR beta cDNA molecules quantification, normalized to 10,000 uniquely labeled [25] TCR beta cDNA molecules by down-sampling, and spectratype plots were compared. As expected [26], the repertoire of 6 years old shows a bell-shaped spectratype with almost no clonal expansions. The repertoire of 64 years old donor reveals several substantially expanded clonotypes highlighting significant changes in T-cell repertoire structure (Fig. 1).

Spectratype can also be used to spot out-of-frame clonotypes and a thorough look at Fig. 1 reveals the abundance of out-of-frame clonotypes in 6 yo sample (small bars corresponding to CDR3 lengths that are not a multiple of 3). Summary report provided by VDJviz shows that the total abundance of out-of-frame clonotypes is ~2 times more for the young donor compared to aged one (*P* < 0.0001, Fisher’s exact test). Out-of-frame TCR sequences are extremely useful for studying V-(D)-J recombination mechanics [27–31] as they are not subject to thymic selection. However, they are relatively rare in mRNA-based samples due to nonsense-mediated mRNA decay. The result shown on Fig. 1 suggests that a deeper sampling is required to detect a sufficient number of out-of-frame clonotypes for repertoires having a high fraction of expanded clonotypes.

### Example 2: variable and joining segment usage

**Fig. 2:**
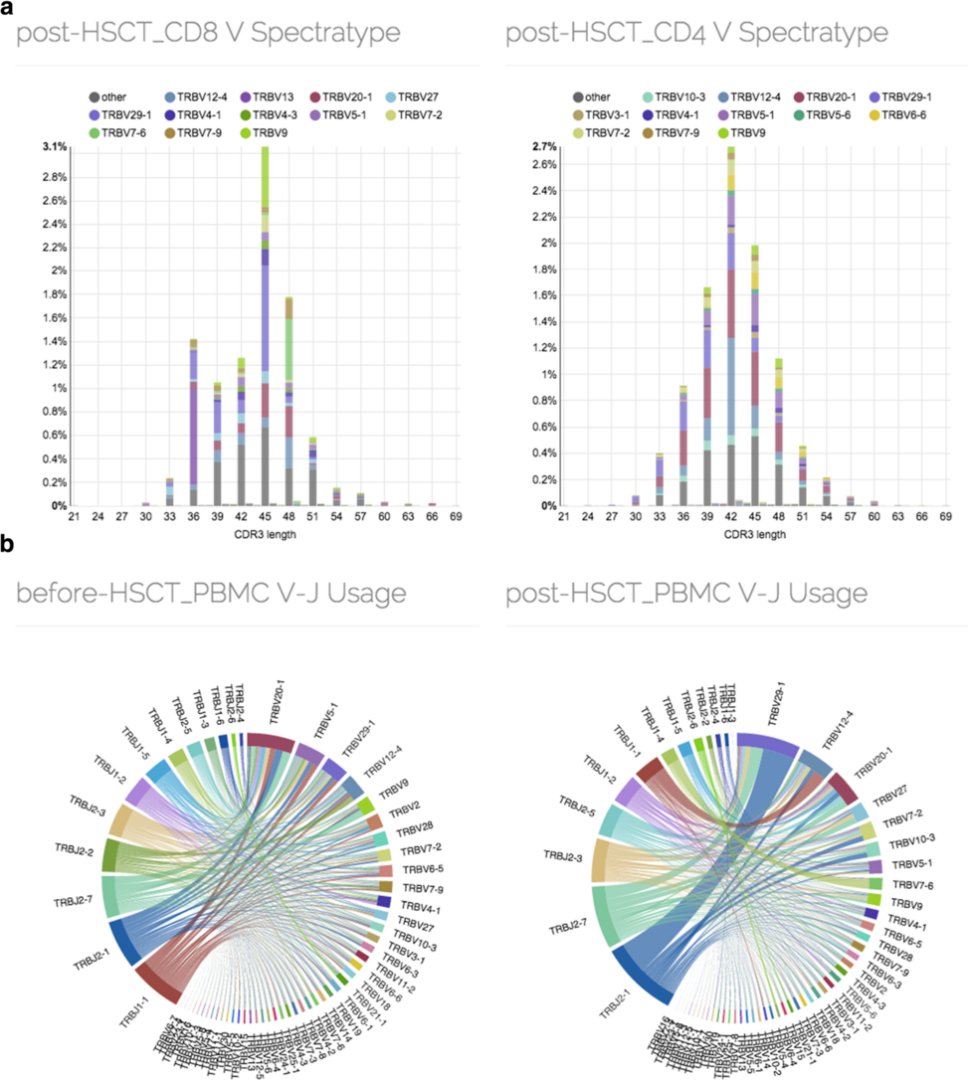
Variable segment spectratype and Variable-Joining segment usage chord diagram. **a** Distribution of CDR3 nucleotide sequence lengths weighted by clonotype frequency. Most enriched Variable segments are explicitly shown. **b** Chord diagram of Variable-Joining junction abundance. Segment lengths are scaled according to the abundance of a specific segment, arc widths scaled by the abundance of corresponding Variable-Joining junctions

The next example shows the analysis of immune receptor segment usage. We have first compared repertoires of helper (CD4) and cytotoxic (CD8) T-cell subsets from a donor that has undergone an autologous hematopoietic stem cell transplantation (HSCT) using V-spectratype, a histogram of clonotypes binned by CDR3 length and Variable segment. It has been previously shown that post-HSCT T-cell repertoire exhibits clonal expansion associated with cytotoxic T-cell response to cytomegalovirus (CMV) [32–34]. As it can be seen from the spectratype shape in Fig. 2a, the clonal expansions are indeed associated with cytotoxic T-cells and result in altered Variable segment usage profile. Variable segment usage profile changes can be also seen from Variable-Joining usage analysis while browsing the bulk PBMC repertoires of the donor before and after HSCT (Fig. 2b).

### Example 3: clonality analysis

**Fig. 3:**
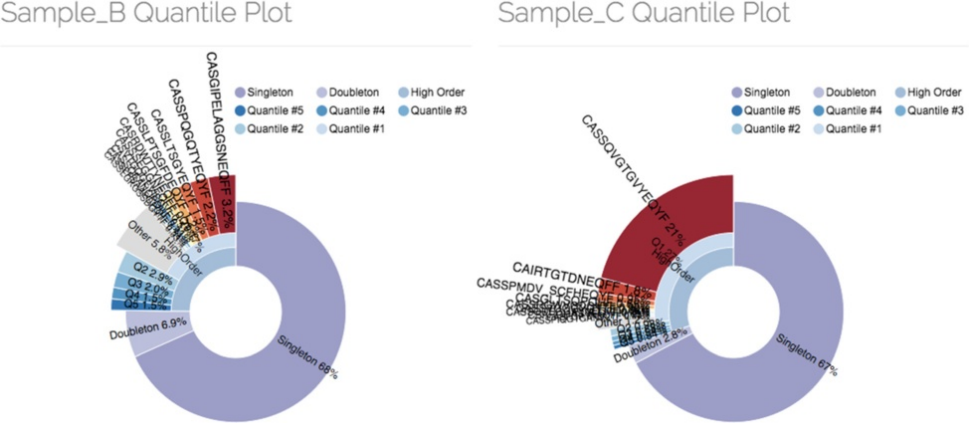
VDJviz clonality plot, a nested pie chart divided into the following regions: singletons (clonotypes represented by a single read), doubletons (2 reads), high order (3 and more reads). High order clonotypes are divided into five quantiles (top 20 % of unique high order clonotypes and so on). Top ten clonotypes of the first quantile are explicitly shown. Size of each segment is the cumulative frequency of all clonotypes that fall into corresponding frequency category

Immune repertoire diversity is one of the key characteristics of the state of adaptive immune system that reflects the ongoing inflammatory processes and defines its ability to effectively mount a response to newly encountered antigens [20]. The following example shows that diversity estimation from Rep-Seq data could be a tricky procedure. For this example we have taken samples, hereafter denoted as B and C, representing repertoires of PBMCs from two healthy female donors of the same age described in Ref. [24]. The samples were normalized to 10,000 uniquely labeled cDNA molecules by down-sampling. The observed diversity computed as the total number of clonotypes is 7425 for sample B and 6967 for sample C, thus B appears to represent a more diverse repertoire. However, closer inspection (Fig. 3) with VDJviz quantile plot feature reveals that sample C has a single dominant clonal expansion, while sample B is characterized by multiple clonal expansions contributing to a heavy tail of clonotype size distribution and effectively having less diversity than sample C. This can be illustrated by calculating Efron-Thisted estimate of total diversity which result in 69,282+/−4480 for sample B and 84,956+/−4488 (23 % higher) for sample C. Therefore, given a sufficient profiling depth the immune repertoire of donor C will turn out to be more diverse than the repertoire of donor B.

### Example 4: rarefaction and error correction

**Fig. 4:**
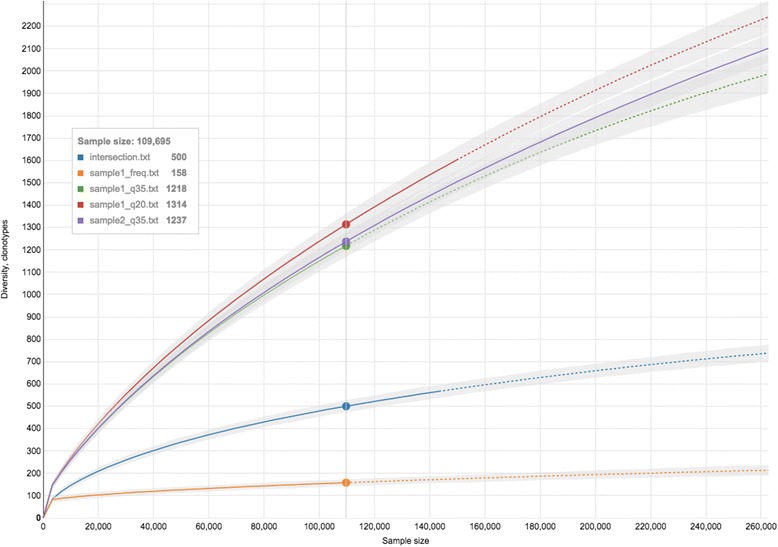
VDJviz interactive rarefaction plot (diversity vs sampling depth) for T-cell repertoires from two replicate PBMC samples processed with various error correction strategies including quality filtering (q20 and q35 thresholds), elimination of clonotypes encountered only in one of the samples (“intersection”) and frequency-based error correction (“freq”). *Solid lines* show rarefaction curves computed using observed clonotype frequencies, *dashed lines* represent their extrapolations. Note that the expected sample diversity is ~90 clonotypes according to UMI-corrected data

Error correction is a critical data processing step in the context of highly complex immune repertoire data. High number of erroneous clonotypes can result in artificial increase of the observed repertoire diversity [12]. Here we compare various error correction methods using rarefaction analysis [35] implemented in VDJviz (Fig. 4). For this purpose two healthy donor PBMCs replicate samples each carrying ~2000 T cells were taken from [36]. Those cDNA libraries were prepared using unique molecular identifier (UMI) tagging approach, and sequenced to a high read-per-UMI coverage allowing nearly complete elimination of PCR and sequencing errors [12]. The correction resulted in the estimate of ~500 TCR beta cDNA molecules and ~90 clonotypes per sample and was used as a gold reference for comparison of various error correction approaches.

First, we tested quality-based filtering (without using UMI information) by removing all clonotypes that have at least one low-quality base (less than Phred quality 20 and 35) in their CDR3 sequence. Such filtering has a relatively small effect on the observed diversity which is more than 10 times higher than the value expected from UMI-corrected results. Notably, using only clonotypes that were found in both samples results in observed diversity that is still ~5 times larger than the expected value, confirming the previous observations that errors that result in artificial diversity are highly reproducible [12]. This indicates that using replicate-based error correction to investigate repertoire diversity [37] is a strategy that should be applied with a great caution. Using frequency-based error correction for quality-filtered sample, namely requiring more than 1:20 abundance ratio difference for merging clonotypes that differ by a single mismatch, showed the best result, yet the observed diversity was still ~60 % higher than the expected value obtained using UMI-correction.

The accuracy and pitfalls of quality- and frequency-based error filtering strategies were previously characterized using a synthetic dataset [9]. As for the comprehensive characterization of the accuracy of UMI-based techniques that are considered as gold-standard in present example, the reader should refer to recently published studies [12, 38].

### Example 5: errors and contamination in repertoire sequencing data

**Fig. 5:**
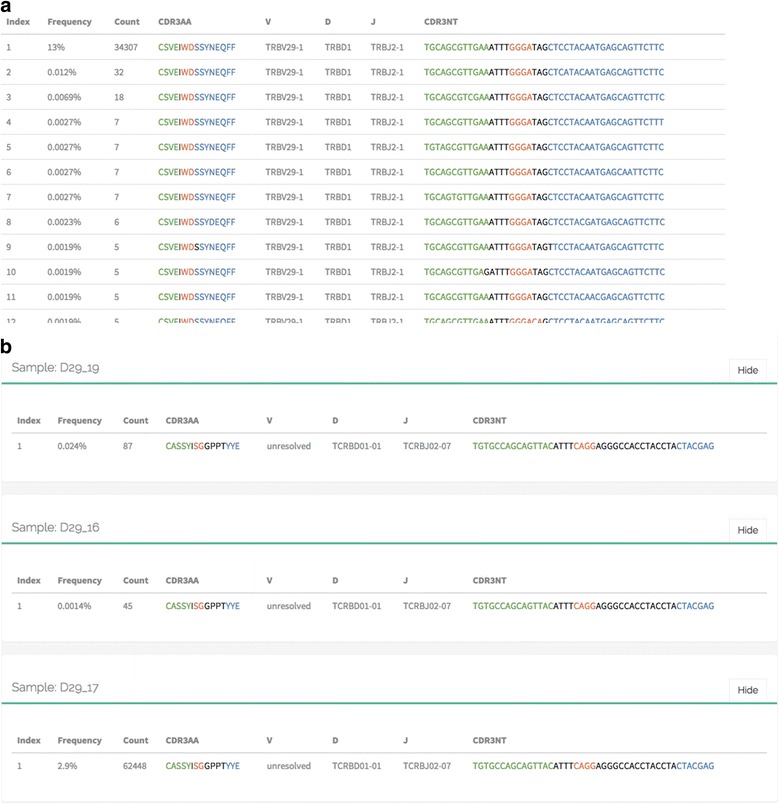
VDJviz clonotype browser interface snapshots showing clonotypes matching a given CDR3 amino acid sequence in a single sample (**a**) and across multiple samples (**b**). **a** A trace of erroneous variants for one of the top clonotypes from sample2_q35 dataset described in example#4. **b** Matching the CDR3 nucleotide sequence of a cancer clonotype in post-treatment samples. The *panel* shows presence of minimal residual disease in corresponding patient (D29_17), as well as cross-sample contamination in two other patients

Our next example demonstrates the clonotype browser engine. In order to visualize the erroneous clonotypes that were the cause of artificial diversity in previous example we have searched for the CDR3 amino acid sequence of the second most abundant clonotype in quality-filtered sample #2. As it could be seen from Fig. 5a, there is a tail of erroneous sub-variants that differ from the real CDR3 nucleotide sequence by a one or more mismatches and are absent in UMI-corrected data. Similar results were obtained for other highly-abundant clonotypes.

Next, we have browsed samples from recently published minimal residual disease (MRD) study [39] to address the issue of cross-sample contamination. In lymphomas, MRD can be monitored by tracking the malignant clonotype sequence in post-treatment immune repertoire. Cross-sample contamination, however, can be a serious issue in this case: contamination with the malignant clonotype which is usually highly abundant can lead to false positive MRD detection. We have checked for the cancer clonotype sequence reported for patient PT-2 in post-treatment samples of other patients (Fig. 5b). Notably, repertoires of 6 out of 42 patients appear to contain exactly the same sequence. This can be hardly explained by coincidence, as no other nucleotide variants were found for the dominant clonotype’s amino acid sequence ruling out convergent recombination. High number of added N-nucleotides in V-D-J junction also supports the fact that CDR3 nucleotide sequence matching in 6 samples simply by chance is highly improbable (*P* < 10^−62^). Notably, the most abundant contamination is present at the level of 24 reads per 100,000 in this example case. Thus, while the method is extremely sensitive and can detect MRD at a level of 1 read per 100,000, such contaminations can severely dampen method’s precision.

### Example 6: public clonotypes

**Fig. 6:**
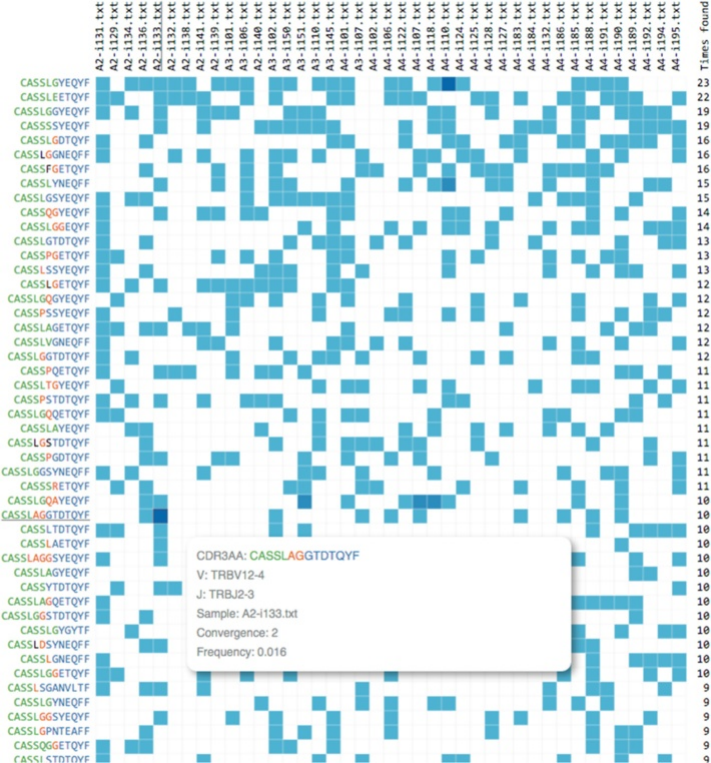
A snapshot of clonotype sharing (Join sample tab of VDJviz) across multiple samples. Clonotypes of 41 healthy donors of various ages were matched by their CDR3 amino acid sequence selecting the ones that were present in at least 10 repertoires

Our last example demonstrates detection of so-called “public” clonotypes, that are a fundamental feature of T-cell repertoire implicated in immune responses to common pathogens and autoimmune responses [40]. We have searched for shared clonotypes in 41 samples each down-sampled to 10,000 uniquely labeled cDNA molecules coming from healthy donors of various ages [24] and required CDR3 amino acid, but not nucleotide, sequence matching in at least 5 of them for a clonotype to be considered “public” (Fig. 6). The total number of unique CDR3 amino acid sequences in those samples was 262,848 and 567 of them represented public clonotypes according to aforementioned criterion. We have next compared our list of clonotypes to data reported by Freeman et al. [41] for a Rep-Seq study of pooled PBMCs coming from 550 individuals of various sex, age and racial background. We found an exactly matching CDR3 amino acid sequences for 127 of clonotypes that we consider “public” (22 %). Many of those clonotypes can be found in other studies (for example, Wang et al. [42]) using Google search engine. That way we have observed 11 of our “public” clonotypes being reported among 29 (excluding “CASSL” which is clearly a non-canonical CDR3 sequence) cancer-specific clonotypes in a recent pancreatic tumor Rep-Seq study [43]. The probability of such overlap occurring by chance is *P* = 2 × 10^−51^ (hypergeometric test, assuming the total number of unique CDR3 amino acid variants is 10^8^ [44]) due to high number of unique CDR3 amino acid variants, highlighting the need for careful statistical testing that account for the presence of clonotypes with a high degree of sharing when dealing with tasks such as tumor-specific clonotype calling. This also suggests that a database of public clonotypes would be a useful resource that can limit the number of false-positives in this case.

While the examples demonstrated here mostly deal with TCR beta sequences, VDJviz can also handle TCR alpha (see Additional file 1: Figure S1), gamma and delta sequences, as well as immunoglobulin sequences (see Additional file 2: Figure S2), albeit with no support for hypermutations in CDR1,2 and framework regions. Overall the examples presented here demonstrate that the analysis modes provided by VDJviz are highly informative and can be used both for explorative analysis and for quality control. The latter is crucial as a multitude of biases can arise due to complexity of Rep-Seq data. While those biases can be dealt with using corresponding techniques or removed manually, their extent should be routinely checked every time an analysis of Rep-Seq data is performed.

While VDJviz web tool can be extended in many ways by adding new analysis types, the most important challenge is to implement intuitive interface for visualizing somatic hypermutations in B-cell repertoires [7] and novel paired-chain Rep-Seq data [45–47].

## Conclusions

As we have demonstrated, VDJviz allows to have a grasp of immune repertoire structure for samples of interest in several clicks and can be easily used by immunologists and biologists with little computational knowledge. VDJviz is not limited to a single library preparation protocol or Rep-Seq processing software including highly popular IMGT HighVQuest [8] and ImmunoSEQ platforms (http://www.adaptivebiotech.com/immunoseq). VDJviz allows great flexibility and can be easily installed as a local server, therefore we believe that in perspective it can become a handy tool-of-choice for immunologists routinely working with immune repertoire data.

## Availability and requirements

• Both standalone and online demo VDJviz versions can be found at https://github.com/antigenomics/vdjviz.

• Operating system(s): platform independent.

• Programming language: Java, JavaScript, Scala.

• Other requirements: Java 1.8, Play Framework.

• License: free for non-profit and academic use.

## Additional files

Additional file 1: Figure S1. Shared TCR alpha CDR3 amino acid sequences in T-regulatory cells reported in Ref. [48]. Note the highlighted clonotype, that is almost exclusively present in wild-type samples, but not CNS3-KO samples that have an altered T-regulatory cell repertoire structure. (TIF 1593 kb) Additional file 2: Figure S2. A representative view of clonotype table from a sequencing experiment involving a hypermutating Raji cell line (our unpublished data) showing CDR3 hypermutations. (TIF 2130 kb)
