# Supplementary figures and images for: VDJviz: a versatile browser for immunogenomics data
Source: BMC Genomics. 2016 Jun 13;17:453. doi: 10.1186/s12864-016-2799-7 (PMC4907000; doi:10.1186/s12864-016-2799-7)

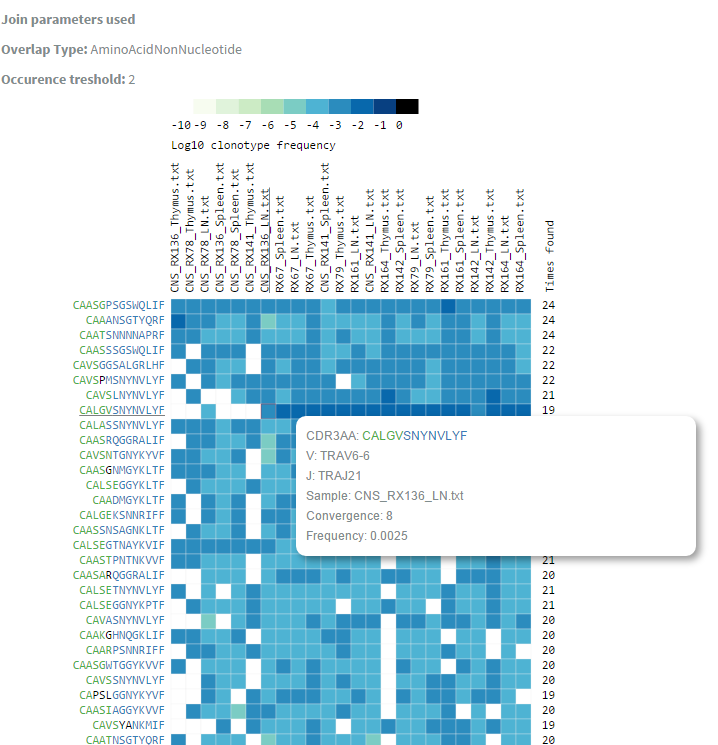

Supplement: Additional file 1: Figure S1. — Shared TCR alpha CDR3 amino acid sequences in T-regulatory cells reported in Ref. [48]. Note the highlighted clonotype, that is almost exclusively present in wild-type samples, but not CNS3-KO samples that have an altered T-regulatory cell repertoire structure. (TIF 1593 kb) [file 12864_2016_2799_MOESM1_ESM.tif]

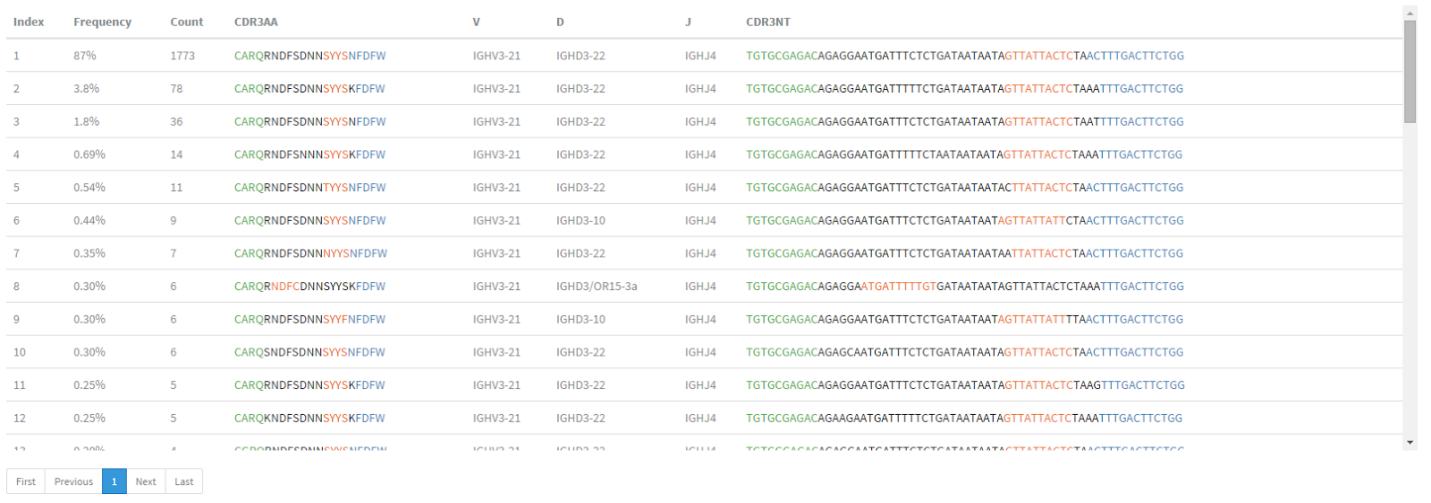

Supplement: Additional file 2: Figure S2. — A representative view of clonotype table from a sequencing experiment involving a hypermutating Raji cell line (our unpublished data) showing CDR3 hypermutations. (TIF 2130 kb) [file 12864_2016_2799_MOESM2_ESM.tif]
